# Leptomeningeal Disease as an Initial Presenting Manifestation in Breast Cancer

**DOI:** 10.7759/cureus.19666

**Published:** 2021-11-17

**Authors:** Jasmine Shrestha, Asis Shrestha, Pratul Karki, Ajay Dhakal

**Affiliations:** 1 Medicine, Nepal Medical College, Kathmandu, NPL; 2 Internal Medicine, Rochester General Hospital, Rochester, USA; 3 Internal Medicine, Bassett Medical Center, Cooperstown, USA; 4 Oncology, University of Rochester Medical Center, Rochester, USA

**Keywords:** brain metastasis, breast cancer, prognosis, leptomeningeal carcinomatosis, abemaciclib

## Abstract

Leptomeningeal disease (LMD) occurs in less than 5% of breast cancer patients. Rarely, patients present with LMD at diagnosis. We report a case of a 59-year-old female who presented with lower back pain, radicular neuropathy with lower extremity weakness, and was found to have diffuse LMD. Workup was initiated to evaluate the etiology of LMD and later involved iliac bone biopsy showed metastatic adenocarcinoma consistent with breast carcinoma. Patient received radiation therapy followed by abemaciclib with letrozole and responded well with the treatment.

## Introduction

Leptomeningeal disease (LMD), also known as leptomeningeal carcinomatosis, refers to infiltration of tumor cells into the leptomeninges (pia and arachnoid mater) or cerebrospinal fluid (CSF) in the arachnoid space. The prevalence of LMD is approximately 10% in patients with metastatic cancer in the course of disease. The three most common solid tumors giving rise to LMD are breast cancer, lung cancer, and melanoma [1]. Leptomeningeal carcinomatosis is seen in about 2-5% of breast cancer patients and carries poor prognosis [2]. In this case report, we present a case of LMD, which was the first presenting manifestation of patient’s breast cancer but had good response to the hormone and radiation therapy.

This case was previously presented as a poster in NYACP Poster Competition on February 29, 2020.

## Case presentation

A 59-year-old female, current smoker with 20 pack-years history, with a past medical history only significant for hypertension, gradually developed anorexia, nausea, fatigue, and weight loss. She initially presented to the emergency department with left flank pain and on CT scan of the abdomen was found to have diffuse osteosclerotic lesions in visualized bones. She was then followed up in primary care clinic where workup for an occult malignancy was initiated. There was no palpable mass or axillary adenopathy on breast examination. She had multiple mammograms in the past, some of which had shown suspicious architecture, which was followed up with multiple breast ultrasounds that had revealed benign findings. Mammogram was repeated and was reported benign with BI-RADS 2. Nuclear bone scan was unremarkable. CT chest revealed no pulmonary lesions but there were small mediastinal, submental, and axillary lymphadenopathy and several subcutaneous lesions on the back (one of which was excised and showed inclusion epidermal cyst). Multiple myeloma workup was negative.

**Figure 1:**
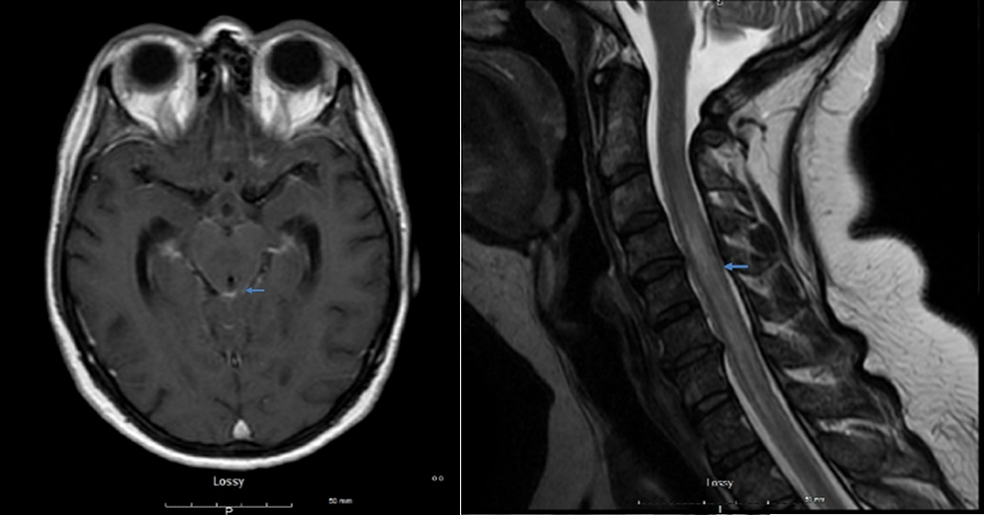
MRI brain and cervical spine showing leptomeningeal enhancement (shown by arrows) at the time of presentation

While the workup was ongoing, the patient started to experience lower back pain associated with weakness of lower extremities, numbness, tingling, and balance issues. She developed constipation as well as urinary incontinence. MRI of the brain and spine redemonstrated similar bony lesions in vertebrae, and also revealed abnormal leptomeningeal enhancement in the brainstem extending along the entire spinal cord (Figure 1). Due to this finding, the patient was admitted to the hospital for further workup. Her mentation was normal. Deep tendon reflexes were absent in lower extremities, Babinski was positive bilaterally, and gait was ataxic. Strength was overall 5/5 in upper extremities and 4/5 in lower extremities. Sensations to touch, pain, temperature, and vibration were normal. Cranial nerve examination was normal, and cerebellar signs were absent. Her thyroid-stimulating hormone was normal. Lumbar puncture showed increased protein (1187 mg/dL) and white blood cells 43 cells/uL (lymphocytes 70%, monocytes 30%) in CSF. CSF cytology was negative for carcinoma and showed numerous lymphocytes but the negative finding could also have been due to delay in processing the specimen. Peripheral blood flow cytometry revealed monoclonal B cell lymphocytosis, non-CLL type. Infectious workups including human immunodeficiency virus, rapid plasmin reagent, Epstein-Barr virus, herpes simplex virus, cytomegalovirus, lyme, and cryptococcal antigen were negative.

**Figure 2:**
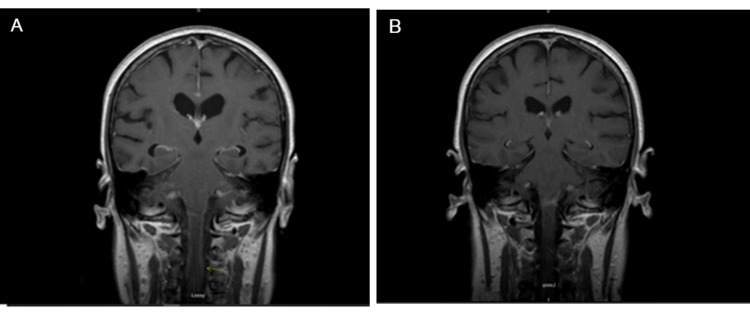
MRI brain and cervical spine showing leptomeningeal enhancement (yellow arrow) at the time of presentation (A) and no leptomeningeal enhancement 2 years after systemic therapy (B)

Finally, right iliac bone biopsy was done that revealed metastatic adenocarcinoma, GCDFP-15 and GATA-3 positive, indicative of breast carcinoma. Estrogen (ER) positive (90%), progesterone (PR) positive (40%), human epidermal growth factor receptor 2 (HER-2)/neu non-amplified IHC 1+, and Ki-67 10%. Tumor markers CA 15-3, CA 27.29, and carcinoembryonic antigen were raised at 85 U/mL, 84.2 U/mL, and 7.1 ng/mL, respectively. She was started with letrozole initially with dexamethasone taper, followed by palliative radiation therapy. The radiation was given to lumbosacral spine with 30 Gy in 10 fractions, which improved her lower back pain. After completion of the radiation therapy, abemaciclib was added to letrozole as the systemic therapy. She showed excellent response to the treatment and her previously seen leptomeningeal enhancements are no longer seen in the follow-up scans (Figure 2). She does continue to have sclerotic changes in her bones; however, she deferred from bone-targeted therapy. The patient has been on abemaciclib and letrozole for about two years, her neurological symptoms have improved, and now she has a good quality of life. She is able to walk half a mile on flat ground and is able to perform light household chores.

## Discussion

Our patient had initially presented with LMD with unknown etiology. Malignancy was suspected but the CSF cytology could not detect malignant cells and no primary lesions were identified. This led to an extensive workup to determine other possible etiologies including infectious and autoimmune, besides malignancy. Bone biopsy was also performed at the involved area, which finally resulted in metastatic adenocarcinoma consistent with breast primary.

LMD occurs in ≤5% of breast cancer patients. It usually occurs at the advanced stage of the disease and may occur as the first presentation of metastasis in 9-15% of patients diagnosed with breast cancer [3]. Rarely, patients can present with LMD at diagnosis without previously known breast cancer as in our case [4,5].

Leptomeningeal metastasis is most common with infiltrating lobular breast carcinoma, and this predisposition is thought due to the changes in cell adhesion molecules found in this subtype [6]. Triple-negative breast cancer (40%) has shown the highest propensity followed by ER/PR positive with HER2 negative molecular subtypes (37%). HER2 positive subtypes, which generally metastasize to the brain parenchyma, involve leptomeninges less frequently [2]. 

LMD in general occurs in the advanced stage and is associated with poor prognosis. Though LMD from breast cancer has better prognosis when compared to LMD from other malignancies, the median overall survival following diagnosis is still four weeks and may be prolonged up to five months with multimodality treatment [6-8]. It is not clear if the survival depends on the tumor subtype. Various studies have attempted to prognosticate based on the hormone receptor status and have obtained different results but no clear consensus has been reached yet. Patients with HER2+ breast cancers have shown better outcomes and those with triple negative (HR- HER2-) have shown the shortest survival after diagnosis in some studies [9,10], whereas those with HR+ status have shown better prognosis in other studies [11,12]. However, the performance status at diagnosis has been consistently proven to be the most important prognostic factor in these patients [2,7].

Current treatment modalities recommended for breast cancer patients with LMD are intrathecal chemotherapy, radiation therapy to the affected area, and approaches to decrease the intracranial pressure [3]. Treatment involves multidisciplinary care with symptom management and pain control, and therapies are given with palliative intent. Since our patient was symptomatic from lumbar radiculopathy and cauda equina syndrome, prompt lumbosacral spine radiotherapy was administered for symptom relief. She was also started on abemaciclib with letrozole for systemic therapy. Abemaciclib is an oral cyclin-dependent kinase (CDK 4/6) inhibitor that has demonstrated progression-free survival and overall survival in advanced HR+/HER2- breast cancers when used in combination with a hormonal therapy [13,14]. It can cross the blood-brain barrier at lower doses and its concentrations in the CSF were found to be comparable to the levels in plasma, which makes it a preferable drug for ER-positive HER2-negative breast cancer with brain metastasis [15,16]. Fortunately, our patient has been showing dramatic response to the therapy with improvement in her clinical symptoms and resolution of imaging findings of the previous leptomeningeal changes.

## Conclusions

LMD is a rare initial presentation of breast cancer. With LMD, there should be a high suspicion for malignancy and should prompt repeated CSF evaluations, neuroaxis imaging, and biopsy of involved lesions to lead to an earlier diagnosis to start treatment. Though LMD in breast cancers has overall poor prognosis, patients with good functional level prior to the diagnosis may have comparatively better outcomes with prompt treatment as seen in our patient. Abemaciclib with hormonal therapy has shown improved progression-free survival and overall survival outcomes which was also observed in our patient.
